# Mitoception: A Novel Strategy to Alleviate Pulmonary Fibrosis

**DOI:** 10.3390/biology15141112

**Published:** 2026-07-09

**Authors:** Sarayu Bhogoju, Parth Patel, Neeraj Kapur, Prashant D. Kunjadia, Ajoy Aloysius, Dave-Preston Esoe, Jamie L. Sturgill, Christine F. Brainson, Luksana Chaiswing, Patrick G. Sullivan, Anthony N. Gerber, Edward Castillo, Stewart F. Graham, Ishanu Chattopadhyay, Girish Nair

**Affiliations:** 1 Department of Internal Medicine, Division of Pulmonary, Critical Care, and Sleep Medicine, University of Kentucky, Lexington, KY 40506, USA; sbh237@uky.edu (S.B.); p.patel0820@outlook.com (P.P.); anthony.gerber@uky.edu (A.N.G.); 2 Department of Internal Medicine, Division of Gastroenterology and Hepatology, University of Kentucky, Lexington, KY 40506, USA; neeraj.kapur@uky.edu; 3 Department of Neuroscience, University of Kentucky, Lexington, KY 40506, USA; pdku223@uky.edu (P.D.K.); patsullivan@uky.edu (P.G.S.); 4 Spinal Cord and Brain Injury Research Center, University of Kentucky, Lexington, KY 40506, USA; 5 Department of Biology, University of Kentucky, Lexington, KY 40506, USA; aaloysius@uky.edu; 6 Department of Toxicology and Cancer Biology, University of Kentucky, Lexington, KY 40506, USA; davepreston.esoe@uky.edu (D.-P.E.); cfbrainson@uky.edu (C.F.B.); l.chaiswing@uky.edu (L.C.); 7 Department of Microbiology, Immunology, and Molecular Genetics, University of Kentucky, Lexington, KY 40506, USA; jamie.sturgill@uky.edu; 8 Department of Biomedical Engineering, The University of Texas at Austin, 107 W Dean Keeton St, Austin, TX 78712, USA; edward.castillo@utexas.edu; 9 Department of Internal Medicine, Corewell Health William Beaumont University Hospital, Royal Oak, MI 48073, USA; stewart.graham@corewellhealth.org; 10 Department of Internal Medicine, Corewell Health Research Institute, Metabolomics Department, Royal Oak, MI 48073, USA; 11 Oakland University William Beaumont School of Medicine, Department of Internal Medicine, Rochester, MI 48309, USA; 12 Department of Internal Medicine-Biomedical Informatics, Sanders-Brown Center on Aging, University of Kentucky, Lexington, KY 40506, USA; ishanu_ch@uky.edu

**Keywords:** pulmonary fibrosis (PF), mitochondrial dysfunction, mitochondrial transfer, mitoception, profibrotic signaling

## Abstract

Pulmonary fibrosis (PF) is a progressive lung condition characterized by irreversible scarring and high mortality, with limited effective treatments. Mitochondrial dysfunction has emerged as a critical factor in fibroblast activation in PF, although approaches to restore mitochondrial function remain underexplored. The present study investigated whether mitochondrial transfer from alveolar type II epithelial cells (A549) to patient-derived fibroblasts could restore mitochondrial function and bioenergetics. Histological analysis of fibrotic lungs reveals increased collagen deposition and elevated profibrotic markers, accompanied by reduced expression of mitochondrial biogenesis and respiratory proteins compared to non-fibrotic controls, indicating mitochondrial impairment. Freshly isolated donor mitochondria were functionally validated before mitoception using Seahorse analysis and patient-derived fibroblasts were confirmed by qRT-PCR using fibroblast-specific markers. In vitro transfer of mitochondria to diseased patient-derived fibroblasts exhibited a modest, dose and time-dependent increase in mitochondrial membrane potential compared to normal fibroblasts. Gene expression analysis revealed decreased fibrosis-associated markers and increased expression of mitochondrial and antioxidant genes following mitoception. Seahorse analysis after mitoception revealed enhanced ATP-linked respiration and improved selected mitochondrial bioenergetic parameters, whereas maximal respiration and spare respiratory capacity demonstrated variable responses. In contrast, normal fibroblasts displayed minimal changes. Collectively, these findings indicate that mitochondrial transfer modulates fibroblast bioenergetics and profibrotic signaling, supporting its potential as a therapeutic strategy for pulmonary fibrosis.

## Simple Summary

Pulmonary fibrosis (PF) is a chronic, progressive fibrotic lung disease. Mitochondrial dysfunction has been identified as a driver for disease progression. This study examined whether the transfer of mitochondria from non-fibrotic alveolar type II epithelial cells (A549) to patient-derived fibroblasts (mitoception) may enhance cellular function. We noted fibrotic lung tissues and diseased fibroblasts exhibit increased profibrotic markers and reduced mitochondrial function. Following mitochondrial transfer, diseased fibroblasts showed increased mitochondrial activity, decreased expression of fibrosis-related genes, and more balanced energy metabolism, whereas normal fibroblasts remained largely unchanged. These findings provide proof-of-concept that epithelial cell-derived mitochondrial transfer can modulate fibroblast bioenergetics and profibrotic signaling.

## 1. Introduction

Pulmonary fibrosis (PF) is a chronic, progressive interstitial lung disease characterized by excessive extracellular matrix deposition, lung architecture distortion, and gradual decline in lung function [1]. Median survival after diagnosis is three to five years, especially with idiopathic pulmonary fibrosis (IPF). While antifibrotic agents including pirfenidone, nintedanib, and nerandomilast effectively reduce the annual rate of forced vital capacity (FVC) decline, they do not reverse established structural lung damage or halt underlying disease progression [2,3,4]. Thus, there is a critical gap in understanding disease mechanisms and a need for newer treatment options. Disrupted communication between epithelial cells and fibroblasts is central to the pathogenesis of PF [5,6]. Repeated alveolar epithelial injury and impaired repair lead to persistent activation of fibroblasts and myofibroblasts, the main sources of extracellular matrix driving fibrosis [7]. In addition to profibrotic signaling, recent evidence shows that metabolic dysregulation and mitochondrial dysfunction are key contributors to fibroblast persistence [8].

Fibroblasts from lungs with PF exhibit impaired energy production, altered mitochondrial function, increased oxidative stress, and a shift toward glycolysis [9,10]. This shift in metabolism probably helps the cells cope with mitochondrial problems, since less oxidative phosphorylation forces fibroblasts to rely more on glycolysis for energy and building blocks. Higher glycolytic activity in these fibroblasts helps them into collagen-producing myofibroblasts, leading to increased cell growth, survival, and extracellular matrix buildup [10]. Importantly, mitochondrial dysfunction can be both a cause and a result of fibrosis, as poor mitochondrial metabolism can activate fibroblasts, and the fibrotic environment can worsen mitochondrial damage and metabolic changes.

Alveolar epithelial cells and fibroblasts sustain lung homeostasis through continuous communication involving soluble factors, vesicles, direct cell contact, and metabolic signals. In pulmonary fibrosis, recurrent epithelial injury disrupts this intercellular crosstalk, resulting in persistent fibroblast activation and excessive extracellular matrix deposition. Although cytokine- and growth factor-mediated communication has been extensively investigated, the contribution of metabolic organelles such as mitochondria remains less well characterized [5,6,7].

Mitochondria are transferred between cells via nanotubes, vesicles, gap junctions, or direct uptake, thereby supporting metabolic recovery in recipient cells. Recent studies have further demonstrated that isolated extracellular mitochondria can be rapidly internalized by recipient cells through active uptake mechanisms, where they contribute thereby contributing to cellular bioenergetics and intracellular signaling [11,12]. In lung injury models, mitochondria derived from stromal or stem cells delivered to damaged epithelial cells restore cellular energy function and promote tissue repair. Recent evidence indicates that mitochondrial transfer or transplantation may attenuate pulmonary fibrosis by reestablishing mitochondrial homeostasis and reducing profibrotic activity [12,13,14,15]. Alveolar type II epithelial cells are responsible for maintaining alveolar structure and interacting with fibroblasts during tissue repair [16]. Their high metabolic activity, mitochondrial abundance, and reparative capacity position them as potential mitochondrial donors [16]. Mitochondrial dysfunction in these cells is associated with impaired repair, aging, and fibrosis [17]; however, it remains uncertain whether these mitochondria directly influence fibroblast metabolism or fibrotic processes.

Although mitochondrial transfer therapy has garnered significant interest, most research has concentrated on transfer from stromal cells to epithelial cells or from exogenous sources [18]. Whether mitochondrial transfer from epithelial cells to fibroblasts modulates fibroblast metabolism, antioxidant defenses, or fibrosis-related signaling remains unknown. In this study, we systematically investigate whether the transfer of mitochondria from alveolar epithelial-like cells to patient-derived pulmonary fibrosis fibroblasts influences mitochondrial membrane potential, cellular respiration, and the expression of fibrosis-associated genes.

## 2. Materials and Methods

### 2.1. Human Lung Tissue Samples

Human lung tissue samples were collected from patients with pulmonary fibrosis who underwent lung transplantation at the University of Kentucky. Normal control lung tissues were obtained from the Kentucky Organ Donor Affiliates (KODA) through donor lungs deemed unsuitable for clinical transplantation and subsequently donated for research. Sample collection was approved by the University of Kentucky Institutional Review Board (IRB) and conducted in accordance with the University of Kentucky’s institutional guidelines and regulations. All tissue samples were procured and stored through the Kentucky Research Alliance for Lung Disease (KRALD) Biobank. Clinical information for the samples included in this study is provided in Supplementary Table S1.

### 2.2. Immunohistology

Human lung tissues were obtained from PF patients and non-fibrotic controls under approved institutional protocols. Formalin-fixed, paraffin-embedded sections were subjected to immunohistochemical staining for fibrotic markers Masson’s Trichrome (Newcomer Supply, Middleton, WI, USA; Cat. No. 9179B), Sirius Red (Abcam, Cambridge, UK; Cat. No. ab150681), and MTCO1 antibody (Invitrogen, Waltham, MA, USA; Cat. No. PA5-79701), as previously described [19,20,21].

### 2.3. Patient-Derived Fibroblasts

Primary fibroblasts were isolated from a non-fibrotic control lung tissue sample (normal fibroblasts; NF) and from a pulmonary fibrosis lung tissue sample (diseased fibroblasts; DF). Briefly, after receiving human lung tissues, samples were weighed, and a portion was finely minced using sterile instruments. For enzymatic digestion, minced tissue was transferred to RPMI-based enzyme medium (supplemented with antibiotics, HEPES, DNase (30 U/mL), collagenase (0.001 g/mL), and dispase (50 U/mL) at a ratio of 10 mL enzyme solution per gram of tissue. This mixture was incubated overnight at room temperature with gentle stirring to generate a single-cell suspension. The next day, the digested tissue was filtered through a 70–100 µm cell strainer. The filtrate was then centrifuged at 1500 rpm for 10 min at 4 °C. After discarding the supernatant, red blood cells were lysed in the pellet using Ammonium–Chloride–Potassium (ACK) buffer for 1 min at room temperature, followed by washing with PBS. The resulting cell pellet was resuspended in complete RPMI medium (Gibco, Thermo Fisher Scientific, Waltham, MA, USA; Cat. No. 11875093) with 10% FBS and, if needed, passed through a strainer to remove debris. Viable cells were counted using trypan blue exclusion and plated in tissue culture dishes. Fibroblasts were isolated based on adherence to plastic; non-adherent cells were removed after 24–48 h. Adherent fibroblasts were maintained in complete medium, with media changes every 2–3 days, and expanded for downstream experiments.

Patient-derived fibroblasts were maintained in RPMI-1640 medium supplemented with GlutaMAX™ (Gibco, Thermo Fisher Scientific, Waltham, MA, USA; Cat. No. 61870036), 10% heat inactivated fetal bovine serum (FBS) (Avantor^®^ Seradigm, VWR International, Radnor, PA, USA; Cat. No. 89510-188), and 1% Antibiotic-Antimycotic (Gibco, Thermo Fisher Scientific, Waltham, MA, USA; Cat. No. 15240062). Plasmocin (InvivoGen, San Diego, CA, USA; Cat. No. ant-mpt) was included at 1% when required. All experiments utilized fibroblasts between passages 3 and 5.

### 2.4. Fibroblast Characterization

The identity of isolated primary cell populations, specifically normal fibroblasts (NF) and diseased fibroblasts (DF), was verified using quantitative real-time PCR (qRT-PCR) and immunofluorescence staining. Fibroblast-associated markers, including α-smooth muscle actin (αSMA), collagen type I alpha 1 (*COL1A1*), vimentin (*VIM*), fibroblast activation protein (*FAP*), and fibronectin 1 (*FN1*), were quantified by qRT-PCR. To exclude epithelial cell contamination, the expression of epithelial markers, including epithelial cell adhesion molecule (*EPCAM*), keratin 8 (*KRT8*), keratin 18 (*KRT18*), and mucin 1 (*MUC1*), were also assessed. The human bronchial epithelial cell line BEAS-2B (ATCC^®^ CRL-9609™, American Type Culture Collection, Manassas, VA, USA) was cultured in Advanced Dulbecco’s Modified Eagle Medium (Advanced DMEM; Gibco, Thermo Fisher Scientific, Waltham, MA, USA; Cat. No. 12491015) supplemented with 10% heat-inactivated fetal bovine serum (FBS; Avantor^®^ Seradigm, Radnor, PA, USA), 1% Antibiotic-Antimycotic (Gibco, Thermo Fisher Scientific, Waltham, MA, USA), 1% GlutaMAX™ (Gibco, Thermo Fisher Scientific, Waltham, MA, USA), and 1% Plasmocin (InvivoGen, San Diego, CA, USA) as required. BEAS-2B cells were used as an epithelial control for fibroblast characterization. The human alveolar epithelial-like cell line A549 (ATCC^®^ CCL-185™, American Type Culture Collection, Manassas, VA, USA) was maintained under identical conditions and served as a positive epithelial control for fibroblast characterization and as the mitochondrial donor for all mitoception experiments. Both cell lines were maintained at 37 °C in a humidified incubator with 5% CO_2_ and were passaged at approximately 75–80% confluence. Relative gene expression was normalized to the housekeeping gene GAPDH and calculated using the 2^−ΔΔCt^ method. The primer sequences used in this study are provided in Table 1.

In addition, immunofluorescence (IF) staining for vimentin (VIM) and fibronectin 1 (FN1) were performed on NF, DF, and A549 cells to further validate the fibroblast phenotype. Briefly, Normal fibroblasts (NF), diseased fibroblasts (DF), and A549 cells were seeded onto chamber slides and cultured until reaching 70–80% confluence. The cells were washed with phosphate-buffered saline (PBS) and fixed with 4% paraformaldehyde for 15 min at room temperature. After fixation, the cells were washed three times with PBS and permeabilized using 0.1% Triton X-100 for 10 min. Blocking was performed with 5% bovine serum albumin (BSA) in PBS for 1 h at room temperature, followed by overnight incubation at 4 °C with primary antibodies against vimentin (R&D Systems, Minneapolis, MN, USA; Cat. No. MAB2105) and fibronectin 1 (Cell Signaling Technology, Danvers, MA, USA; Cat. No. 26836S). After primary antibody incubation, the cells were washed three times with PBS and incubated for 1 h at room temperature, protected from light, with Alexa Fluor 488 goat anti-rat IgG and Alexa Fluor 594 goat anti-rabbit IgG secondary antibodies (1:200 dilution). Nuclei were counterstained with ProLong Gold Antifade Mountant containing DAPI (Thermo Fisher Scientific, Waltham, MA, USA). Fluorescence images were captured using identical exposure settings for all experimental groups. Supplementary Figure S1 presents representative images and the antibodies used in IF are listed in Table 2.

### 2.5. Mitochondrial Isolation and Mitoception

Mitochondria were separated from cell pellets using mechanical disruption followed by differential centrifugation. A549 alveolar epithelial-like cells were selected as the mitochondrial donor source because they are a well-established human alveolar type II epithelial cell model, widely utilized in pulmonary fibrosis and lung epithelial biology research [22,23]. These cells facilitate reproducible mitochondrial isolation, demonstrate stable growth characteristics, and provide sufficient mitochondria for mitoception experiments. A549 cells were cultured until they reached approximately 75–80% confluency, followed by trypsinization and mitochondrial isolation.

Mitochondria were isolated from A549 cells by differential centrifugation using a protocol adapted from previously published methods with minor modifications [24,25]. The mitochondrial isolation buffer (MIB) was made with 215 mM mannitol, 75 mM sucrose, 0.1% (*w*/*v*) bovine serum albumin (BSA), 20 mM HEPES, and 1 mM EGTA. The pH was set to 7.2 with KOH. The buffer was prepared in ultrapure water and kept cold on ice during the process to protect the mitochondria. All subsequent steps were carried out on ice or at 4 °C. Cell pellets were resuspended in 500 µL of ice-cold MIB and pipetted gently to avoid creating air bubbles. The mixture was moved to a 2 mL microcentrifuge tube and homogenized with four strokes using a 26-gauge needle (Becton, Dickinson and Company (BD), Franklin Lakes, NJ, USA). The volume was adjusted to 2 mL with MIB, then centrifuged at 1300× *g* for 3 min at 4 °C to remove nuclei and debris. The supernatant was moved to a new tube and spun at 13,000× *g* for 10 min at 4 °C to collect crude mitochondria. The resulting pellet was resuspended in 500 µL MIB and homogenized with four strokes using a 28-gauge needle. The volume was brought up to 2 mL with MIB, centrifuged at 1300× *g* for 3 min at 4 °C, and the supernatant was spun again at 13,000× *g* for 10 min at 4 °C to get the final mitochondrial pellet. The final mitochondrial pellet was resuspended in 50 µL MIB and kept on ice for immediate use. Protein concentration was measured with a BCA assay before starting further experiments.

### 2.6. Donor Mitochondrial Quality Assessment

To verify the integrity and functionality of the isolated A549 donor mitochondria prior to mitoception, mitochondrial bioenergetics were assessed immediately after isolation using a Seahorse XFe96 Flux Analyzer (Agilent Technologies, Santa Clara, CA, USA). Freshly isolated A549 mitochondria were loaded into Seahorse XFe96 cell culture microplates at protein concentrations of 3, 5, 7.5, 10, and 15 µg per well. Oxygen consumption rate (OCR) was measured using a mitochondrial stress test protocol, as previously described [26]. Briefly, State III respiration, representing ADP-stimulated oxidative phosphorylation, was measured following sequential injection of pyruvate (5 mM), malate (2.5 mM), and ADP (4.3 mM) (Port A). State IV respiration, representing proton leak-dependent respiration, was determined following injection of oligomycin (2.5 µM; Port B). State V (Complex I) respiration was measured after addition of FCCP (4 µM; Port C), while State V (Complex II) respiration was determined following sequential injection of rotenone (0.8 µM) and succinate (10 mM; Port D). Oxygen consumption rates were continuously monitored throughout the assay to evaluate mitochondrial respiratory function.

To assess the functional quality of the isolated donor mitochondria, mechanically damaged mitochondria were included as a negative control. Mechanically damaged mitochondria were generated by subjecting freshly isolated mitochondrial preparations to five repeated freeze–thaw cycles in liquid nitrogen immediately before Seahorse analysis and were used as a negative control. As previously reported, damaged mitochondria exhibit substantially reduced respiratory activity compared with intact mitochondria [27]. Each experimental condition was analyzed using five technical replicates, and the experiment was repeated using three independent biological replicates. Donor mitochondria were isolated from 10,000 A549 cells, and oxygen consumption rate (OCR) measurements were normalized to the corresponding donor cell number. Data are presented as mean ± SEM. Statistical analyses were performed using GraphPad Prism version 10.0 (GraphPad Software, San Diego, CA, USA). Differences among mitochondrial concentrations and treatment groups were analyzed using two-way analysis of variance (ANOVA) followed by Tukey’s multiple-comparison test, with *p* < 0.05 considered statistically significant.

### 2.7. Mitoception

Freshly isolated mitochondria from A549 cells were quantified using the Pierce™ BCA Protein Assay Kit (Thermo Fisher Scientific, Waltham, MA, USA) immediately after isolation. Based on the measured protein concentration, mitochondria were diluted in serum-free RPMI-1640 medium to final concentrations of 5 μg or 15 μg per treatment. Patient-derived normal fibroblasts (NF) and diseased fibroblasts (DF) were seeded 24–36 h before mitoception to achieve approximately 70–80% confluency at the time of treatment. Prior to mitochondrial transfer, the culture medium was replaced with serum-free RPMI-1640 medium containing the appropriate amount of freshly isolated mitochondria. Untreated cells receiving serum-free medium alone served as negative controls. Serum-free medium was used during mitoception to minimize interference from serum proteins and to facilitate efficient interaction between donor mitochondria and recipient fibroblasts.

Cells were incubated with isolated mitochondria for 3 h, 6 h, or 24 h at 37 °C in a humidified incubator with 5% CO_2_. Following the treatment periods, the mitochondrial suspension was carefully removed, and complete growth medium was added. Cells were then incubated overnight before subsequent analyses. Following mitoception, cells were processed for downstream analyses, including mitochondrial membrane potential, mitochondrial mass, mitochondrial reactive oxygen species (mtROS), quantitative RT-PCR and Seahorse extracellular flux analysis. All mitoception experiments were performed using three independent biological replicates, with technical replicates included for each assay as described in the corresponding methods sections.

### 2.8. qRT-PCR

Total RNA was isolated from the frozen lung tissue samples and cultured cells using the RNeasy Mini Kit (Qiagen, Germantown, MD, USA; Cat. No. 74104). RNA concentration and purity were determined using a NanoDrop™ spectrophotometer (Thermo Fisher Scientific, Waltham, MA, USA). The reverse transcriptase reaction was performed with High-Capacity cDNA Reverse Transcription Kit (Applied Biosystems, Thermo Fisher Scientific, Waltham, MA, USA; Cat. No. 4368814). Expression of genes of interest was determined by RT-PCR on an ABI Step One Plus Real Time system using Power SYBR green PCR master mix (Applied Biosystems, Thermo Fisher Scientific, Waltham, MA, USA; Cat. No. A25742). Primers were designed by Primer Express software 3.0 (Applied Biosystems). Relative gene expressions were normalized to the appropriate housekeeping gene based on the experimental assay. *GAPDH* was used for qRT-PCR characterization of A549, BEAS-2B, and patient-derived fibroblasts, whereas *β-actin* was used for lung tissue qRT-PCR and mitoception experiments. All assays were performed in triplicate. Relative expression level fold changes were calculated using the 2^−ΔΔCt^ method. Primer sequences for all target and housekeeping genes are provided in Table 1.

### 2.9. Western Blotting

Total protein was extracted from frozen human lung tissue samples and cultured fibroblasts using RIPA lysis buffer (Thermo Fisher Scientific, Waltham, MA, USA; Cat. No. 89900) supplemented with protease and phosphatase inhibitors according to a previously described protocol [28] and BCA assay. Lysates were incubated on ice for 30 min with intermittent vertexing and centrifuged at 12,000× *g* for 15 min at 4 °C. The supernatant was collected, and protein concentration was determined using the Pierce™ BCA Protein Assay Kit (Thermo Fisher Scientific, Waltham, MA, USA; Cat. No. 23225). Equal amounts of protein (30–50 μg) were resolved on 4–20% SDS-PAGE gels (Thermo Fisher Scientific, Waltham, MA, USA) and transferred onto Immobilon^®^-FL PVDF membranes (MilliporeSigma, Burlington, MA, USA) using the Power Blotter XL Semi-Dry Transfer System (Invitrogen™, Thermo Fisher Scientific, Waltham, MA, USA). Membranes were blocked with Pierce™ Protein-Free Blocking Buffer (Thermo Fisher Scientific, Waltham, MA, USA) for 1 h at room temperature and incubated overnight at 4 °C with primary antibodies diluted 1:1000 in blocking buffer. Following three washes with TBST (Tris-buffered saline containing 0.1% Tween-20), membranes were incubated with the appropriate HRP-conjugated secondary antibodies for 1 h at room temperature. Membranes were washed again, and protein bands were visualized using West Femto Maximum Sensitivity Chemiluminescent Substrates (Thermo Fisher Scientific, Waltham, MA, USA; Cat. No. 34094) according to the manufacturer’s instructions. Band intensities were quantified using ImageJ software (1.54t) (National Institutes of Health, Bethesda, MD, USA) and normalized to the β-actin loading control.

Primary antibodies used in this study included α-smooth muscle actin (αSMA; Cell Signaling Technology, Danvers, MA, USA; Cat. No. 19245S), fibronectin 1 (FN1; Cell Signaling Technology; Cat. No. 26836S) and transforming growth factor-β (TGFβ; Cell Signaling Technology; Cat. No. 3709S). Complete antibody information used in this study is provided in Table 2.

### 2.10. Mitochondrial Potential

Normal or diseased fibroblasts were seeded at 10,000 cells per well in 96-well plates and incubated for 36 to 48 h to allow attachment. Cells were then treated with mitochondria at 5 µg and 15 µg concentrations for 3 h and 24 h time points; untreated cells served as controls (n = 20 technical replicates per group).

Following the treatment, mitochondrial membrane potential was assessed using MitoTracker Red CMXRos dye (Thermo Fisher Scientific, Waltham, MA, USA; Cat. No. M46752). A 150 nM working solution was freshly prepared in pre-warmed HBSS, and 100 µL was added to each well. Cells were incubated at 37 °C for 15 to 20 min, protected from light, and washed gently 2–3 times with HBSS. Fluorescence was measured using a plate reader (Ex/Em: 579/599 nm). Fluorescence intensity indicated mitochondrial membrane potential. Data are presented as mean ± SEM from three independent biological experiments.

Holotomography imaging was performed using a Tomocube HT-X1 (Tomocube Inc., Daejeon, Republic of Korea) microscope to visualize early mitochondrial association with recipient fibroblasts following mitoception, as previously described [29]. Recipient fibroblasts were stained with MitoTracker™ Red CMXRos according to the manufacturer’s instructions. Holotomographic images were captured 10 min after the addition of isolated donor mitochondria. Representative three-dimensional refractive index and fluorescence images were collected to assess the early localization of mitochondrial signals in recipient fibroblast cells. Fluorescence intensity was quantified using ImageJ software (National Institutes of Health, Bethesda, MD, USA), with identical image acquisition and analysis parameters applied across all experimental groups.

### 2.11. Mitochondrial Mass

Mitochondrial mass was quantified using MitoTracker™ Green FM (Thermo Fisher Scientific, Waltham, MA, USA; Cat. No. M46750), a membrane potential-independent fluorescent probe that selectively labels mitochondria. Normal or diseased fibroblasts were seeded at a density of 10,000 cells per well in 96-well plates and incubated for 36–48 h. Cells were treated with mitochondria (5 µg and 15 µg) for 3 h and 24 h, while untreated cells served as a control (n = 20 technical replicates per group). Following treatment, mitochondrial mass was assessed using MitoTracker Green FM dye (Thermo Fisher Scientific, M46750). A 200 nM working solution was prepared fresh in pre-warmed HBSS. Cells were incubated with 100 µL per well of the dye solution at 37 °C for 20 min protected from light. After incubation, cells were gently washed 2–3 times with warm HBSS to remove excess dye. Fluorescence was measured using a plate reader (Ex/Em: 490/516 nm) Fluorescence intensity was used as a measure of mitochondrial mass. Data are presented as mean ± SEM from three independent biological experiments.

### 2.12. Mitochondrial ROS

Mitochondrial superoxide levels were measured using the MitoSOX Red indicator (Thermo Fisher Scientific, Waltham, MA, USA; M36008). Normal and diseased lung fibroblasts were seeded in a 96 well plates and incubated for 36–48 h. Cells were treated with isolated mitochondria at concentrations of 5 µg and 15 µg in serum-free medium, followed by replacement with complete medium. Untreated cells served as controls (n = 20 technical replicates per group). At 3 h and 24 h post-treatment, cells were washed with pre-warmed HBSS and incubated with MitoSOX Red at a final concentration of 200 nM in serum-free medium for 10 min at 37 °C, protected from light. Following incubation, cells were washed three times with warm HBSS to remove excess dye. Fluorescence was measured immediately using a plate reader or imaging system with excitation and emission wavelengths of approximately 510 and 580 nm, respectively. Data are presented as mean ± SEM from three independent biological experiments.

### 2.13. Seahorse Analysis

A mitochondrial stress test was performed using the Seahorse XFe96 Flux Analyzer (Agilent Technologies, Palo Alto, CA, USA) according to the manufacturer’s instructions [30]. Patient-derived fibroblasts were seeded at a density of 10,000 cells per well in a Seahorse XFe 96 Assay Plate (Agilent, Santa Clara, CA, USA) and incubated for 24 to 36 h. Following incubation, cells were treated with mitochondria isolated from epithelial alveolar type II cells at concentrations of 5 μg and 15 μg for either 3 h or 24 h. After mitoception cells were incubated in normal growth media overnight, they were analyzed using Seahorse. On the day of the assay, Seahorse XF Assay Medium (Agilent, Santa Clara, CA, USA; 102365) was supplemented with 10 mM glucose, 1 mM pyruvate, and 2 mM glutamine. Cells were washed twice with XF Assay Medium and then incubated for one hour in 175 μL of XF Assay Medium in a humidified chamber at 37 °C with atmospheric CO_2_. After incubation, oxygen consumption rate (OCR) and extracellular acidification rate (ECAR) were measured at 37 °C using the Seahorse XFe 96 Analyzer (Agilent, Santa Clara, CA, USA). During the assay, treatments were injected sequentially to achieve the following final concentrations: 1.0 μM oligomycin (Biomol, Hamburg, Germany; CM-111), 4 μM carbonyl cyanide-p-trifluoromethoxyphenylhydrazone (FCCP) (Biomol, Hamburg, Germany; CM120), 10 mM succinate (MilliporeSigma, Burlington, MA, USA; S-7501) with 0.8 μM rotenone (Biomol, Hamburg, Germany; CM-117), and 1 μM antimycin A (MilliporeSigma, Burlington, MA, USA; A8674) and 2-deoxyglucose (2DG). OCR values were normalized to the average basal rate among all treatments per 1000 cells for each experiment. Specific rates were calculated based on equations provided by Agilent Biosciences (Santa Clara, CA, USA), including basal, oligomycin, FCCP, rotenone/succinate, antimycin A, ATP-linked, maximum, reserve, complex I, complex II, leak, non-mitochondrial, and mitochondrial area under the curve (AUC) OCRs. Glycolysis, glycolytic capacity, and glycolytic reserve were determined using normalized ECAR data. Each experimental group consisted of 20 technical replicates, and all experiments were independently repeated three times. Data are presented as mean ± SEM.

### 2.14. Statistical Analysis

Data are presented as mean ± standard error of the mean (SEM). All statistical analyses were performed using GraphPad Prism version 10.0 (GraphPad Software, San Diego, CA, USA). All experiments were independently repeated three times. Comparisons between two groups were analyzed using an unpaired two-tailed Welch’s *t*-test. Comparisons among three or more groups were analyzed using an ordinary one-way analysis of variance (ANOVA), followed by Tukey’s multiple comparisons test for post hoc analysis. Comparisons involving two independent variables were analyzed using a two-way ANOVA, followed by Tukey’s multiple comparisons test. Homogeneity of variance was assessed using the Brown–Forsythe and Bartlett’s tests where appropriate. A *p*-value < 0.05 was considered statistically significant.

## 3. Results

### 3.1. Pulmonary Fibrosis Lung Tissues Exhibit Enhanced Fibrotic Remodeling and Reduced Mitochondrial Markers

**Figure 1:**
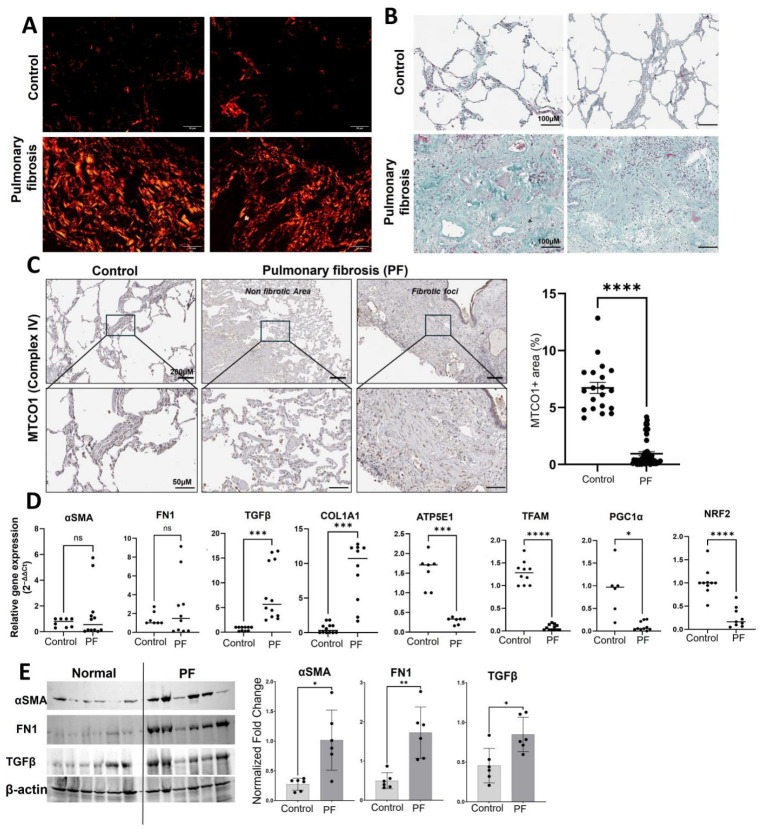
Pulmonary fibrosis is associated with increased fibrosis and reduced mitochondrial biogenesis in human lung tissues. (**A**) Representative Sirius Red staining of normal control and pulmonary fibrosis (PF) lung tissue sections showing collagen deposition. Scale bar = 50 µm. (**B**) Representative Masson’s Trichrome staining of normal control and PF lung tissue sections. Scale bar = 100 µm. (**C**) Representative immunohistochemical staining for mitochondrial cytochrome c oxidase subunit I (MTCO1), with corresponding quantitative analysis. Scale bar = 200 µm; inset scale bar = 50 µm. (**D**) Relative mRNA expression (2^−ΔΔCt^) of fibrosis-associated and mitochondrial biogenesis-associated genes in normal and PF lung tissues. (n = 8–12 biological samples per group) (**E**) Representative Western blot analysis of fibrosis-associated proteins with β-actin as the loading control, together with corresponding densitometric quantification, (n = 6 biological samples per group. Data are presented as mean ± SEM). Statistical analysis was performed using an unpaired two-tailed Welch’s *t*-test. * *p* < 0.05; ** *p* < 0.01; *** *p* < 0.001; **** *p* < 0.0001; ns, not significant.

To establish the disease-associated molecular phenotype, fibrotic and control human lung tissue explants were examined by histology, immunohistochemistry, qRT-PCR, and Western blot. Sirius Red and Trichrome staining demonstrated extensive collagen deposition in pulmonary fibrosis tissue compared with control tissue, confirming the presence of tissue remodeling (Figure 1A,B). In parallel, immunostaining for the mitochondrial complex IV marker MTCO1 was reduced in PF tissues, indicating loss of mitochondrial signal in diseased tissue (Figure 1C). These tissue-level observations were supported by mRNA analyses showing significant upregulation of profibrotic markers, including *αSMA*, *FN1*, *COL1A1* and *TGFβ*, in pulmonary fibrosis samples relative to controls (Figure 1D). In contrast, markers associated with mitochondrial biogenesis, *TFAM*, *PGC1α*, and *NRF2*, were decreased in fibrotic lungs (Figure 1D). Western blot analysis further supported these findings, showing increased profibrotic protein levels in pulmonary fibrosis tissue (Figure 1E). Collectively, these data indicate that pulmonary fibrosis lungs are characterized by both exaggerated profibrotic signaling and impaired mitochondrial homeostasis.

### 3.2. Patient-Derived Pulmonary Fibroblasts Exhibit Characteristic Fibroblast Features

**Figure 2:**
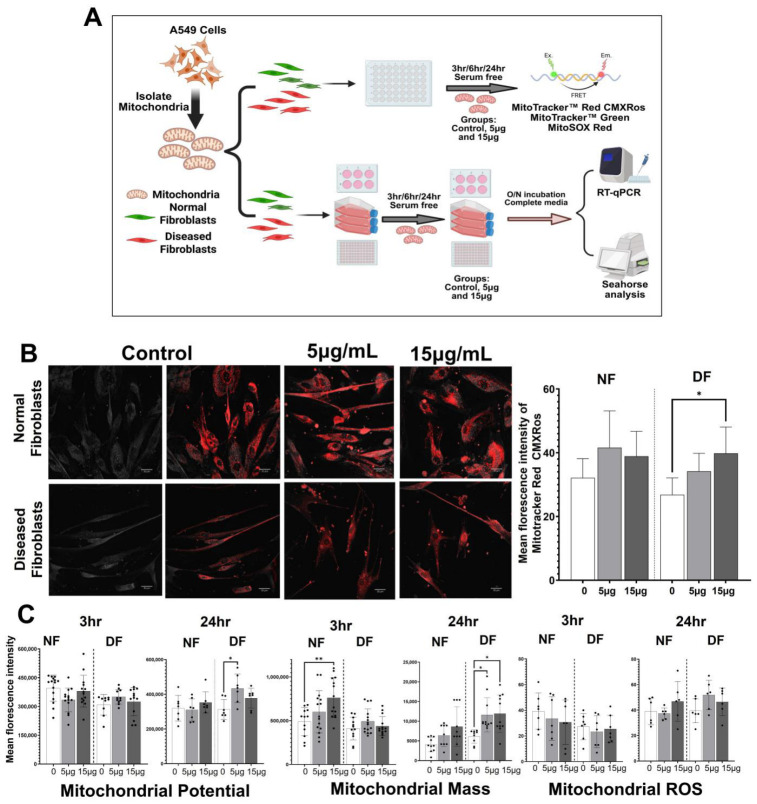
Mitoception enhances mitochondrial membrane potential and mitochondrial mass in patient-derived pulmonary fibroblasts. (**A**) Schematic illustration of the mitoception workflow showing the transfer of A549-derived mitochondria into normal fibroblasts (NF) and diseased fibroblasts (DF), Link for the Schematic: https://app.biorender.com/illustrations/620f20932eecad00534cb0f5, accessed on 29 June 2026. (**B**) Representative holotomography images acquired 10 min after mitoception showing early mitochondrial association with recipient fibroblasts following staining with MitoTracker™ Red CMXRos. Corresponding fluorescence intensity quantification is shown. Scale bar = 30 µm. (**C**) Quantification of mitochondrial membrane potential using MitoTracker™ Red CMXRos, mitochondrial mass using MitoTracker™ Green FM, and mitochondrial superoxide using MitoSOX™ Red in NF and DF treated with 5 µg or 15 µg donor mitochondria for 3 h and 24 h. Data are presented as mean ± SEM from three independent biological replicates, each performed with 20 technical replicates. Statistical analysis was performed using an ordinary one-way ANOVA followed by Tukey’s multiple-comparisons test. * *p* < 0.05; ** *p* < 0.01; Non-significant comparisons are not shown.

Given that pulmonary fibrosis tissues exhibited reduced mitochondrial content together with increased fibrotic remodeling, we next established patient-derived fibroblast models to investigate whether restoration of mitochondrial function through mitoception could attenuate the fibrotic phenotype. The experimental details are as shown in Figure 2A.

The identity of the isolated primary cell populations was confirmed by assessing the expression of fibroblast- and epithelial-associated markers using qRT-PCR and IF (Supplementary Figure S1A,B). Compared to A549 cells, both NF and DF exhibited substantially higher expression of fibroblast markers. Specifically, *αSMA* expression increased by approximately 230-fold (*p* < 0.001) in NF and 280-fold (*p* < 0.001) in DF, while *COL1A1* expression increased by approximately 220-fold (*p* < 0.001) in NF and 160-fold (*p* < 0.01) in DF. *FN1* and *VIM* expression levels were also significantly elevated (approximately 1.5-fold (*p* < 0.0001) and 1.6-fold (*p* < 0.0001), respectively), and *FAP* expression increased by approximately 3500- to 3700-fold (*p* < 0.05) in both fibroblast populations relative to A549 cells. In contrast, epithelial markers *EPCAM*, *KRT8*, *KRT18*, and *MUC1* were highly expressed in A549 cells but showed minimal expression in NF and DF, indicating negligible epithelial cell contamination.

Immunofluorescence analysis further validated the fibroblast phenotype by demonstrating strong VIM and FN1 staining in both NF and DF, whereas only minimal staining was observed in A549 cells (Supplementary Figure S1B). Collectively, these findings confirm the successful isolation and characterization of patient-derived pulmonary fibroblasts for subsequent mitoception experiments.

### 3.3. Donor Mitochondria Remain Functionally Active Following Isolation

The functional integrity of donor mitochondria prior to mitoception was assessed by measuring mitochondrial respiration immediately after isolation using the Seahorse XFe96 Extracellular Flux Analyzer (Supplementary Figure S1C). Intact mitochondria demonstrated a dose-dependent increase in State III (ADP-stimulated) respiration, indicating preserved ATP-producing capacity. State V Complex I and State V Complex II respiration also increased with mitochondrial protein concentration, demonstrating robust electron transport chain activity following isolation. In contrast, mitochondria subjected to repeated freeze–thaw cycles exhibited markedly reduced oxygen consumption across all respiratory states, confirming a substantial loss of mitochondrial function. While State IV respiration increased modestly at higher mitochondrial concentrations, the significantly lower respiratory activity in damaged mitochondria compared with intact preparations confirmed successful preservation of mitochondrial bioenergetic function during the isolation procedure. Consequently, mitochondrial protein concentrations of 5 μg and 15 μg were selected for subsequent mitoception experiments, as these concentrations retained robust respiratory activity and provided sufficient mitochondrial material for downstream functional analyses.

### 3.4. Mitoception Increases Mitochondrial Membrane Potential in Diseased Fibroblasts

To determine whether epithelial cell-derived mitochondria could improve mitochondrial function in fibroblasts, isolated A549 mitochondria were transferred into normal fibroblasts (NF) and diseased fibroblasts (DF), and mitochondrial membrane potential was assessed using MitoTracker Red CMXRos at 3 and 24 h after treatment. Holotomography demonstrated early mitochondrial signal localization around recipient fibroblasts within 10 min of mitoception, suggesting rapid association of donor mitochondria with recipient cells as indicated by increased MitoTracker Red CMXRos fluorescence (Figure 2B). At the early 3 h time point, only modest changes were observed, suggesting that immediate mitochondrial functional recovery after transfer was limited. However, at 24 h, mitochondrial membrane potential increased more clearly, particularly in diseased fibroblasts (Figure 2C). The effect appeared more evident with mitochondrial treatment than in untreated controls, and the higher mitochondrial dose showed a stronger trend toward improvement. These findings suggest that mitoception does not simply produce an acute transient signal but rather promotes a time-dependent recovery in mitochondrial polarization, especially in fibroblasts derived from diseased lungs. Because diseased fibroblasts begin from a more compromised metabolic state, they appear to derive greater benefit from mitochondrial supplementation than normal fibroblasts.

### 3.5. Mitoception Alters Mitochondrial Mass but Not Mitochondrial ROS

MitoTracker™ Green FM staining was performed to determine whether mitoception altered mitochondrial content in recipient fibroblasts. At 3 h, mitochondrial mass showed only modest changes in both normal fibroblasts (NF) and diseased fibroblasts (DF) following mitochondrial supplementation. By 24 h, mitochondrial mass increased in both NF and DF, with the most pronounced increase observed in DF treated with 15 μg donor mitochondria (Figure 2C). These findings indicate that mitoception promotes a time-dependent increase in mitochondrial content, particularly in diseased fibroblasts.

Mitochondrial ROS. Mitochondrial reactive oxygen species (mtROS) were evaluated using the MitoSOX™ Red indicator. At both 3 h and 24 h, mitoception did not produce significant changes in mitochondrial superoxide levels in either NF or DF compared with untreated controls (Figure 2C). These results suggest that supplementation with epithelial-derived mitochondria did not induce detectable mitochondrial oxidative stress under the experimental conditions.

### 3.6. Mitoception Reduces Profibrotic Gene Expression and Enhances Mitochondrial and Antioxidant Markers

**Figure 3:**
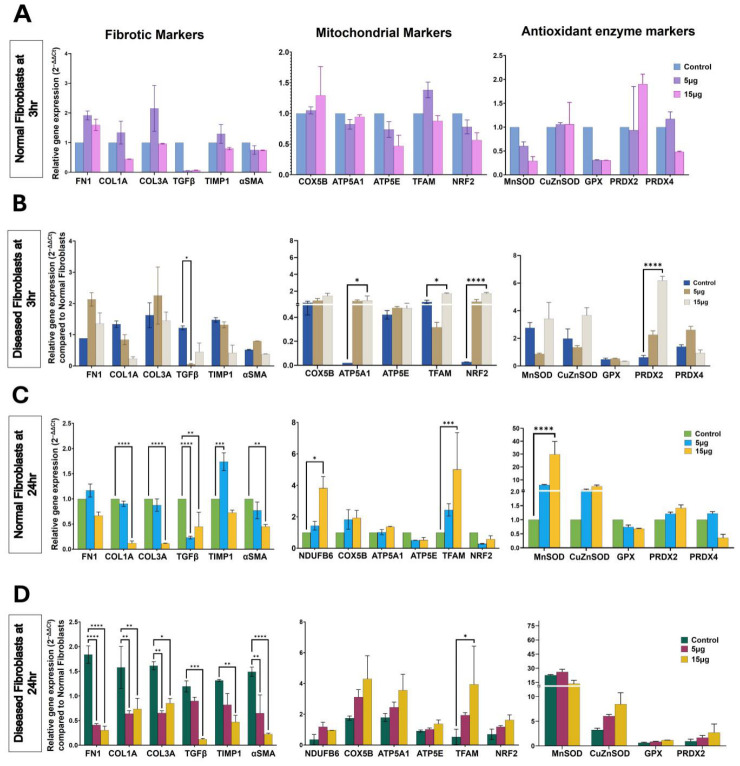
Mitoception modulates the expression of fibrosis-associated, mitochondrial, and antioxidant genes in patient-derived pulmonary fibroblasts. (**A**,**B**) Relative mRNA expression (2^−ΔΔCt^) of fibrosis-associated, mitochondrial, and antioxidant genes in normal fibroblasts (NF) (**A**) and diseased fibroblasts (DF) (**B**) following treatment with donor mitochondria (5 µg or 15 µg) for 3 h. (**C**,**D**) Relative mRNA expression (2^−ΔΔCt^) of fibrosis-associated, mitochondrial, and antioxidant genes in NF (**C**) and DF (**D**) following treatment with donor mitochondria (5 µg or 15 µg) for 24 h. Gene expression was normalized to β-actin. Data are presented as mean ± SEM from three independent biological replicates. Statistical analysis was performed using two-way ANOVA followed by Tukey’s multiple-comparisons test. * *p* < 0.05; ** *p* < 0.01; *** *p* < 0.001; **** *p* < 0.0001. Non-significant comparisons are not shown.

To further investigate the biological effects of mitoception, the expression of profibrotic, mitochondrial, and antioxidant genes was evaluated in normal fibroblasts (NF) and diseased fibroblasts (DF) at 3 h and 24 h following mitochondrial supplementation (Figure 3). At 3 h, mitoception induced only modest transcriptional changes in NF (Figure 3A). In contrast, DF exhibited an early increase in mitochondrial-associated genes, with *ATP5A1* increasing by approximately 1.5-fold (*p* < 0.05), *TFAM* by approximately 2-fold (*p* < 0.05), and *NRF2* by approximately 1.8-fold (*p* < 0.0001) following treatment with 15 μg of mitochondria (Figure 3B). Antioxidant genes also showed an early response, with *PRDX2* increasing approximately 6-fold (*p* < 0.0001), suggesting activation of mitochondrial stress-response pathways.

By 24 h, the effects of mitoception were more pronounced (Figure 3C,D). In DF, profibrotic markers, including *FN1*, *COL1A1*, *COL3A1*, *TGFβ*, *TIMP1*, and *αSMA*, were significantly reduced following mitochondrial supplementation, with the 15 μg treatment producing the greatest effect. Conversely, mitochondrial genes were upregulated, with *TFAM* increasing by approximately 4-fold (*p* < 0.05), indicating enhanced mitochondrial biogenesis and respiratory gene expression. Antioxidant responses were also modulated, with *CuZnSOD* showing the greatest induction following mitoception. In NF, mitochondrial treatment produced comparatively modest changes in fibrotic gene expression but increased *NDUFB6* (*p* < 0.05), *TFAM* (*p* < 0.001), and *MnSOD* (*p* < 0.0001), suggesting that mitoception preferentially enhances mitochondrial homeostasis while exerting a stronger antifibrotic effect in diseased fibroblasts.

At the 6 h time point (Supplementary Figure S3), transcriptional responses were intermediate between those observed at 3 h and 24 h. In NF, mitoception reduced the expression of several profibrotic markers while modestly increasing mitochondrial and antioxidant gene expression. In DF, mitochondrial supplementation significantly decreased collagen-associated genes (*COL1A1* and *COL3A1*; *p* < 0.0001)) and increased mitochondrial genes, including *NDUFB6*, *ATP5A1*, *TFAM*, and *NRF2*, supporting a progressive, time-dependent restoration of mitochondrial homeostasis and suppression of the profibrotic phenotype.

### 3.7. Mitoception Promotes Mitochondrial Fusion in Fibroblasts

To further evaluate the impact of mitoception on mitochondrial dynamics, the mRNA expression levels of mitochondrial fusion markers (*OPA1* and *MFN1*) and fission markers (*DRP1* and *MFF*) were quantified in NF and DF at 3 h and 24 h following treatment with isolated mitochondria (5 µg and 15 µg).

In NF, mitoception significantly increased *OPA1* (*p* < 0.0001) expression at both 3 h and 24 h (Supplementary Figure S3A). At 3 h, *OPA1* levels were markedly elevated in response to both 5 µg and 15 µg mitochondrial treatments, with the strongest induction in the 5 µg group. At 24 h, *OPA1* expression remained significantly elevated, particularly in the 15 µg group. *MFN1* expression showed a modest increase, which became more pronounced at 24 h in the 15 µg group. In contrast, *DRP1* and *MFF* expression exhibited minimal or no significant changes following mitoception. These findings suggest that mitochondrial transfer preferentially promotes mitochondrial fusion over fission in NF.

In DF, mitoception induced a more pronounced response (Supplementary Figure S3B). *OPA1* expression was upregulated at both 3 h and 24 h following mitochondrial transfer, with the greatest increase (*p* < 0.0001) in the 15 µg group. At 3 h, *OPA1* exhibited a strong, dose-dependent increase that persisted at 24 h. *MFN1* expression showed only modest changes relative to *OPA1*. The fission marker *MFF* was significantly reduced at 3 h, while DRP1 expression remained relatively unchanged. By 24 h, *DRP1* and *MFF* levels were largely stable across treatment groups.

Collectively, these findings indicate that mitoception primarily enhances mitochondrial fusion pathways, particularly by upregulating *OPA1*, while exerting minimal effects on mitochondrial fission markers. This effect was more pronounced in DF, suggesting that mitochondrial transfer may contribute to the restoration of mitochondrial network integrity and homeostasis in fibrotic cells.

### 3.8. Mitoception Improves Mitochondrial Respiration in Diseased Fibroblasts

**Figure 4:**
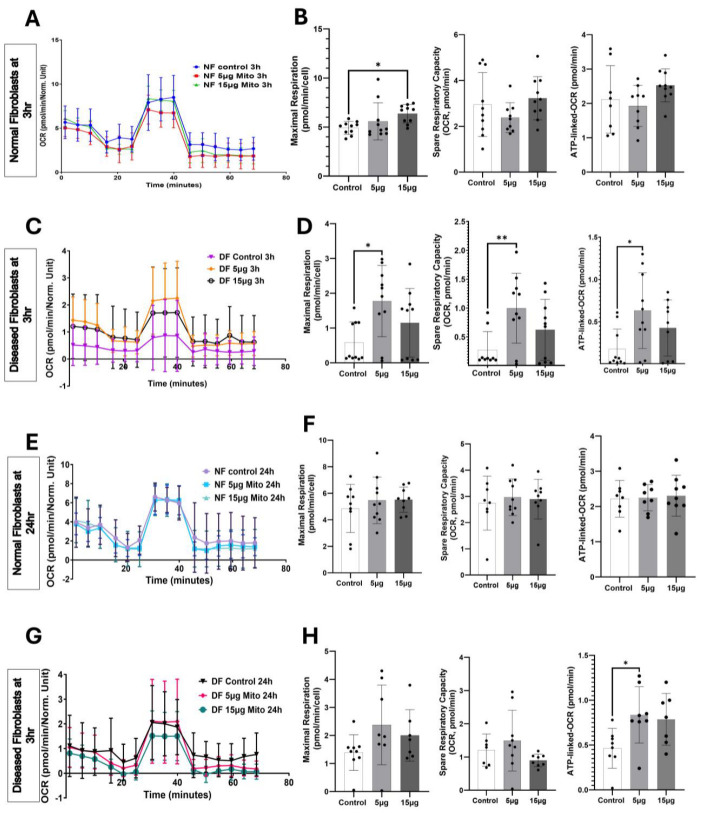
Mitochondrial transfer improves mitochondrial respiration in fibroblasts. (**A**–**D**) Seahorse XFe96 extracellular flux analysis of normal fibroblasts (NF) and diseased fibroblasts (DF) following mitoception for 3 h, showing representative oxygen consumption rate (OCR) traces and quantification of maximal respiration, spare respiratory capacity, and ATP-linked respiration. (**E**–**H**) Seahorse analysis of NF and DF following mitoception for 24 h, showing representative OCR traces and quantification of maximal respiration, spare respiratory capacity, and ATPlinked respiration. Cells were analyzed following overnight recovery in complete growth medium after mitoception. Data are presented as mean ± SEM from three independent biological replicates, each performed with 20 technical replicates. Statistical analysis was performed using ordinary one-way ANOVA followed by Tukey’s multiple-comparisons test. * *p* < 0.05; ** *p* < 0.01; Non-significant comparisons are not shown.

Given that mitochondrial dysfunction is a hallmark of pulmonary fibrosis, mitochondrial respiration was evaluated using Seahorse extracellular flux analysis at 3 h and 24 h following mitoception (Figure 4A–H). At 3 h, mitoception produced only modest changes in NF, with a slight increase in maximal respiration following treatment with 15 μg donor mitochondria, whereas ATP-linked respiration and spare respiratory capacity remained largely unchanged. In contrast, DF exhibited a greater early bioenergetic response, with 5 μg mitochondrial treatment significantly increasing maximal respiration, spare respiratory capacity, and ATP-linked respiration, indicating enhanced mitochondrial respiratory function shortly after mitochondrial supplementation.

By 24 h, the bioenergetic response differed between NF and DF. NF continued to show only minor alterations in OCR parameters, whereas DF demonstrated a significant increase (*p* < 0.05) in ATP-linked respiration following treatment with 5 μg donor mitochondria. Although maximal respiration and spare respiratory capacity showed variable responses across treatment groups, the increase in ATP-linked respiration suggests improved efficiency of oxidative phosphorylation rather than a generalized increase in mitochondrial respiratory capacity. Collectively, these findings indicate that mitoception preferentially enhances mitochondrial energy production in diseased fibroblasts.

To determine whether mitoception also altered glycolytic metabolism, extracellular acidification rate (ECAR) was measured at both 3 h and 24 h (Supplementary Figure S4A,B). No significant differences in basal glycolysis, glycolytic capacity, or glycolytic reserve were observed among treatment groups in either NF or DF, indicating that mitoception primarily improves mitochondrial oxidative metabolism without inducing a detectable shift toward glycolysis.

## 4. Discussion

Intercellular mitochondrial transfer has been extensively investigated in mesenchymal stem cells (MSCs), where donor mitochondria restore bioenergetics and promote tissue repair following lung injury [12,15,18,31]. In contrast, the potential role of epithelial-derived mitochondria in regulating fibroblast metabolism and activation has received limited attention. The current study extends these findings by showing that epithelial-derived mitochondria are associated with enhanced mitochondrial function and reduced profibrotic gene expression in patient-derived pulmonary fibroblasts. These results indicate a previously underexplored mechanism of epithelial fibroblast metabolic communication in pulmonary fibrosis. Overall, these findings provide proof of concept that epithelial-to-fibroblast mitoception can modulate mitochondrial homeostasis and support further investigation of this approach as a potential therapeutic strategy for pulmonary fibrosis.

At the tissue level, fibrotic lungs exhibited increased expression of profibrotic markers, including *αSMA*, *FN1*, and *TGFβ*, together with reduced expression of mitochondrial regulators such as *PGC-1α*, *TFAM*, and *NRF2*. Our findings are consistent with previous evidence suggesting that mitochondrial dysfunction contributes to fibroblast activation and fibrosis progression [9,10,32]. Reduced mitochondrial biogenesis and impaired oxidative phosphorylation have been associated with elevated reactive oxygen species (ROS) production and activation of profibrotic pathways, including TGFβ signaling [33,34]. The observed imbalance between profibrotic signaling and mitochondrial homeostasis further supports the hypothesis that mitochondrial dysfunction acts as a driver, rather than merely a consequence, of fibrosis.

A major finding of this study is that mitochondrial transfer increased mitochondrial membrane potential, particularly in diseased fibroblasts. The increase in mitochondrial membrane potential suggests improved mitochondrial polarization following mitoception. Although holotomography demonstrated early mitochondrial association with recipient fibroblasts, the present study does not directly demonstrate intracellular incorporation or long-term persistence of donor mitochondria. Therefore, the observed improvement in membrane potential should be interpreted as enhanced mitochondrial function associated with mitoception rather than definitive evidence of donor mitochondrial integration. Previous studies have demonstrated that isolated mitochondria can be rapidly internalized by recipient cells through active uptake mechanisms, supporting the biological plausibility of mitochondrial uptake following mitoception [11]. This effect was most evident at 24 h, suggesting that mitoception is a time-dependent process involving mitochondrial uptake, intracellular trafficking, and integration into the host mitochondrial network. Similar intercellular mitochondrial transfer events have been reported in lung injury and other disease models [12,15,35]. The stronger response in DF suggests that metabolically compromised cells are more responsive to mitochondrial supplementation, which is a critical consideration for therapeutic targeting.

In addition to improvements in mitochondrial membrane potential, mitochondrial mass was significantly increased following mitoception. The increase in mitochondrial mass observed after mitoception suggests an increase in mitochondrial content within recipient fibroblasts. Together with the improved membrane potential, these findings indicate enhanced mitochondrial homeostasis following mitochondrial supplementation. This observation aligns with emerging evidence that mitochondrial health, including membrane potential and respiratory efficiency, is critical for cellular function and homeostasis [36].

At the transcriptional level, mitoception significantly reduced the expression of key profibrotic genes, including *αSMA*, *COL3A1*, *FN1*, and *TGFβ*, in DF. These results indicate that mitochondrial transfer not only restores bioenergetic function but also modulates fibroblast phenotype. The reduction in profibrotic signaling may reflect alterations in mitochondrial metabolism and redox-sensitive signaling pathways. Concurrently, the upregulation of mitochondrial and antioxidant genes, such as *TFAM*, *ATP5A1*, *NDUFB6*, and *NRF2*, suggests activation of endogenous mitochondrial repair and stress response mechanisms. *NRF2* is a critical regulator of antioxidant defense and has been implicated in protection against fibrosis [37,38,39]. Therefore, mitoception may exert its effects through both direct metabolic rescue and activation of protective signaling pathways. Although early transcriptional responses were observed following mitoception, alternative mechanisms, including residual components of the mitochondrial preparation or donor-derived signaling molecules, cannot be completely excluded. Because this study was designed as a proof-of-concept investigation, future studies incorporating vehicle controls, mitochondrial-depleted preparations, and pharmacological or genetic inhibition of mitochondrial uptake will be important to confirm that the observed biological responses are directly mediated by transferred mitochondria.

Functional bioenergetic analyses further support these findings. Seahorse data indicate that mitochondrial transfer alters respiratory parameters in diseased fibroblasts, resulting in increased ATP production despite reduced maximal respiration and spare respiratory capacity. This pattern suggests a shift toward more efficient ATP generation under basal conditions, although the capacity to respond to increased energetic demand remains limited. This phenotype likely reflects partial restoration of mitochondrial function, where transferred mitochondria support immediate energy requirements but do not fully re-establish mitochondrial reserve capacity. This observation is consistent with the understanding that mitochondrial dysfunction in pulmonary fibrosis is multifactorial and may require sustained or repeated interventions for complete restoration [40]. The lack of a major ECAR response further suggests that mitoception primarily affects mitochondrial oxidative metabolism rather than inducing a compensatory shift in glycolytic flux. Improved ATP-linked respiration in diseased fibroblasts may indicate more efficient coupling between substrate oxidation, electron transport, and ATP synthesis. From a redox perspective, this is important because inefficient electron transfer can increase NADH pressure, alter the NADH/NAD^+^ ratio, and promote electron leak to oxygen. The increase in ATP-linked respiration suggests more efficient oxidative phosphorylation following mitoception. However, additional studies measuring NADH/NAD^+^ balance and electron transport efficiency are required to determine the mechanisms underlying these bioenergetic changes. However, the incomplete recovery of maximal respiration and spare respiratory capacity indicates that mitochondrial reserve remains limited. This pattern is consistent with partial metabolic rescue: mitoception improves basal energetic and redox function but does not fully restore the capacity of diseased fibroblasts to respond to increased energetic demand [41].

Another notable observation was that mitoception exerted minimal effects on normal fibroblasts. This selectivity suggests potential therapeutic advantages. Mitochondrial transfer appears to preferentially benefit diseased cells while largely preserving normal cellular function. The limited response in normal fibroblasts may be due to their relatively intact mitochondrial networks and bioenergetic status. This could restrict the integration or functional contribution of exogenous mitochondria. Building on these observations, it is important to consider potential limitations. Despite the promising nature of these findings, several limitations should be acknowledged. First, the study is primarily utilized in in vitro models. The efficiency, stability, and long-term effects of mitochondrial transfer in vivo remain uncertain. Furthermore, the mitochondria utilized in the transfer experiments were isolated from A549 cells, a cancer-derived alveolar epithelial-like cell line. While A549 cells are commonly used as an in vitro model of alveolar type II epithelial cells, their metabolic properties may not fully reflect those of primary epithelial cells. Consequently, future research employing primary alveolar epithelial cells or more physiologically relevant models is necessary to further substantiate these findings.

Second, although improvements in mitochondrial markers and bioenergetic profiles were observed, direct assessments of oxidative stress-related damage, such as 8-hydroxy-2′-deoxyguanosine (8-OHdG) and 4-hydroxy-2-nonenal (4-HNE), were not performed. Incorporating these analyses would strengthen the mechanistic link between mitochondrial restoration and antifibrotic effects. Third, the long-term persistence, integration, and functional activity of transferred mitochondria at the protein level were not evaluated. In addition, although mitochondrial biogenesis-related genes were evaluated, protein-level validation of mitochondrial dynamics regulators such as MFN1, MFN2, OPA1, and DRP1 were not performed. Future studies should determine whether the transcriptional changes observed after mitoception are accompanied by remodeling the mitochondrial network. Functional assays evaluating collagen deposition, fibroblast migration, and contractility were beyond the scope of the present study but will be important for determining whether the observed molecular changes translate into functional antifibrotic effects.

Please note, the 24 h increase in mitochondrial membrane potential after mitoception may reflect a redox-relevant restoration of mitochondrial coupling in DF. Improved polarization suggests that transferred mitochondria may contribute to a more functional proton motive force and enhanced oxidative phosphorylation capacity. However, increased membrane potential can also increase the thermodynamic pressure for electron leak if electron flow through the respiratory chain remains impaired. Therefore, the absence of a strong or consistent MitoSOX signal is important, as it suggests that mitochondrial transfer did not produce a major increase in mitochondrial superoxide under the experimental conditions tested [42]. This finding supports the interpretation that mitoception improves mitochondrial function without imposing an additional oxidative burden. Nevertheless, MitoSOX primarily reports mitochondrial superoxide-related oxidation and does not fully capture hydrogen peroxide flux, thiol oxidation, glutathione redox balance, lipid peroxidation, or compartment-specific redox signaling. Thus, the increase in mitochondrial membrane potential should be interpreted as evidence of consistency with improved bioenergetic status, with future studies needed to define how mitochondrial transfer alters the broader mitochondrial redox network.

Importantly, this work should be considered a proof-of-concept study demonstrating the feasibility and biological effects of epithelial-derived mitochondrial transfer in patient-derived pulmonary fibroblasts. To overcome these limitations, future research should employ more physiologically relevant systems, including three-dimensional (3D) culture models and ex vivo lung explant-based mitoception approaches, which more accurately replicate the native tissue microenvironment and cellular interactions. In addition, comprehensive mechanistic studies are necessary to elucidate the molecular pathways underlying mitochondrial uptake, intracellular trafficking, retention, and functional integration, as well as their downstream effects on fibroblast activation, metabolic remodeling, and profibrotic signaling in pulmonary fibrosis. From a translational perspective, these findings highlight mitochondrial transfer as a novel therapeutic approach for pulmonary fibrosis. Enhancing mitochondrial function in fibroblasts may provide a strategy to reverse or attenuate fibroblast activation, a central driver of fibrosis. Additionally, elucidating the mechanisms of epithelial–fibroblast mitochondrial crosstalk could yield new insights into disease progression and reveal further therapeutic targets. Approaches such as engineered mitochondria, extracellular vesicle-mediated mitochondrial delivery, or pharmacological activation of mitochondrial biogenesis may further expand the clinical potential of this strategy.

## 5. Conclusions

This study demonstrates that pulmonary fibrosis is associated with pronounced mitochondrial dysfunction alongside enhanced profibrotic signaling in lung tissue and fibroblasts. We show that mitochondrial transfer from epithelial cells into fibroblasts (mitoception) leads to a functional improvement in mitochondrial activity, as evidenced by increased mitochondrial membrane potential and enhanced ATP production. Importantly, mitoception suppresses key profibrotic markers while upregulating mitochondrial and antioxidant pathways, indicating a shift toward a less activated fibroblast phenotype. These effects are more pronounced in diseased fibroblasts, highlighting the selective benefit of mitochondrial supplementation in metabolically compromised cells. Although mitochondrial transfer partially restores bioenergetic function, incomplete recovery of maximal respiration and spare capacity suggests that additional mechanisms contribute to persistent mitochondrial impairment. Overall, this proof-of-concept study demonstrates that epithelial-derived mitoception is associated with improved mitochondrial homeostasis and reduced profibrotic signaling in patient-derived pulmonary fibroblasts. While additional mechanistic, functional, and in vivo studies are required, these findings establish a foundation for exploring mitochondrial transfer as a potential therapeutic strategy for pulmonary fibrosis.

## Figures and Tables

**Table 1:** List of primers used for gene expression studies.

Primer	Sequence (F)	Sequence (R)
FN1	ACA ACA CCG AGG TGA CTG AGA C	GGA CAC AAC GAT GCT TCC TGA G
COL1A1	GAT TCC CTG GAC CTA AAG GTC C	AGC CTC TCC ATC TTT GCC AGC A
COL3A1	TGT TCT GCA AGG AAT GCC TGG A	TCT TTC CCT GGG ACA CCA TCA G
TGFB1	CAA GCA GAG TAC ACA CAG CAT	TGC TCC ACT TTT AAC TTG AGC C
TIMP1	GGA GAG TGT CTG CGG ATA CTT C	GCA GGT AGT GAT GTG CAA GAG TC
αSMA	CTA TGC CTC TGG ACG CAC AAC T	CAG ATC CAG ACG CAT GAT GGC A
NDUFB6	CTG CAG CAG CTG CGA GA	GAA TAA TCC AGA CAG GTA CAA G
COX5B	TGG CTT CAA GGT TAC TTC GC	AGT CGC CTG CTC TTC ATC AG
ATP5A1	TTT GGG TTC ATC TTT CAT TGC	AAG ACA CGC CCA GTT TCT TC
ATP5E	TAC AGC ATG GTG GCC TAC TG	TCTT CAG TGC ATC TCT CAC TGC
TFAM	ATG CTT ATA GGG CGG AGT GG	TGG TTT CCT GTG CCT ATC CA
NRF2	CAC ATC CAG TCA GAA ACC AGT GG	GGA ATG TCT GCG CCA AAA GCT G
MnSOD	CAG CAT AAC GAT CGT GGT TT	ACA GGC CTT ATT CCA CTG CT
CuZnSOD	GGA AAA GGT GGA AAT GAA GA	GGG CCT CAG ACT ACA TCC AA
GPX	ACG ATG TTG CCT GGA ACT TT	GAT GTC AAG CTC GAT GTC AA
PRDX2	CGA GCA TGG GGA AGT TTG TC	GGC ACA AGC TCA CTA TCC GT
PRDX4	AGA GGA GTG CCA CTT CTA CG	GGA GGT CTT CGC TTT GCT TAG GT
EPCAM	GGCTCTTTAAGGCCAAGCAG	CACTCGCTCAGAGCAGGTTAT
VIMENTIN	GCAAAGATTCCACTTTGCGT	GAAATTGCAGGAGGAGATGC
FAP	GGAAGTGCCTGTTCCAGCAAT	TGTCTGCCAGTCTTCCCTGAAG
KRT8	ACAAGGTAGAGCTGGAGTCTCG	AGCACCACAGATGTGTCCGAGA
KRT18	GCTGGAAGATGGCGAGGACTTT	TGGTCTCAGACACCACTTTGCC
MUC1	CCTACCATCCTATGAGCGAGTAC	GCTGGGTTTGTGTAAGAGAGGC
β-Actin	GATTACTGCTCTGGCTCCTAGC	GACTCATCGTACTCCTGCTTGC
GAPDH	GTCTCCTCTGACTTCAACAGCG	ACCACCCTGTTGCTGTAGCCAA

**Table 2:** List of Antibodies used.

Target	Application	Host Species	Supplier	Catalog No.	Dilution	Detection
MTCO1	Immunohistochemistry	Rabbit	Invitrogen, Thermo Fisher Scientific (Waltham, MA, USA)	PA5-79701	1:150	HRP/DAB
α-Smooth Muscle Actin (αSMA)	Western blot	Rabbit	Cell Signaling Technology (Danvers, MA, USA)	19245S	1:1000	HRP-conjugated Goat Anti-Rabbit IgG (1:5000)
Fibronectin 1 (FN1)	Western blot	Rabbit	Cell Signaling Technology (Danvers, MA, USA)	26836S	1:1000	HRP-conjugated Goat Anti-Rabbit IgG (1:5000)
Transforming Growth Factor-β (TGFβ)	Western blot	Rabbit	Cell Signaling Technology (Danvers, MA, USA)	3709S	1:1000	HRP-conjugated Goat Anti-Rabbit IgG (1:5000)
β-Actin	Western blot	Rabbit	Cell Signaling Technology (Danvers, MA, USA)	4967	1:1000	HRP-conjugated Goat Anti-Rabbit IgG (1:5000)
Vimentin (VIM)	Immunofluorescence	Rat	R&D Systems (Minneapolis, MN, USA)	MAB2105	1:200	Alexa Fluor™ 488 Goat Anti-Rat IgG (1:200)
Fibronectin 1 (FN1)	Immunofluorescence	Rabbit	Cell Signaling Technology (Danvers, MA, USA)	26836S	1:200	Alexa Fluor™ 594 Goat Anti-Rabbit IgG (1:200)

## Data Availability

The original contributions presented in this study are included in the article/Supplementary Materials. Further inquiries can be directed to the corresponding author.

## Supplementary Materials

The following supporting information can be downloaded at: https://www.mdpi.com/article/10.3390/biology15141112/s1, Supplementary Figure S1: Characterization of patient-derived pulmonary fibroblasts and functional validation of donor mitochondria prior to mitoception. (A) qRT-PCR analysis of fibroblast-associated markers (αSMA, COL1A1, FN1, VIM, and FAP) and epithelial markers (EPCAM, KRT8, KRT18, and MUC1) in BEAS-2B cells, A549 cells, normal fibroblasts (NF), and diseased fibroblasts (DF). Gene expression was normalized to GAPDH and expressed as relative mRNA expression (2^−ΔΔCt^) relative to BEAS-2B cells. (B) Representative immunofluorescence images of A549 cells, NF, and DF stained for vimentin (green) and fibronectin 1 (FN1; red), with nuclei counterstained using DAPI (blue). Scale bar = 100 µm. (C) Functional assessment of donor mitochondria immediately following isolation using the Seahorse XFe96 Extracellular Flux Analyzer. Oxygen consumption rate (OCR) was measured at increasing mitochondrial protein concentrations (3, 5, 7.5, 10, and 15 µg) using intact and mechanically damaged mitochondria generated by repeated freeze–thaw cycles. State III (ADP-stimulated respiration), State IV (oligomycin-induced respiration), State V CI (FCCP-stimulated Complex I respiration), and State V CII (succinate-driven Complex II respiration in the presence of rotenone) are shown and Statistical significance represents comparisons between intact and mechanically damaged mitochondria at each mitochondrial protein concentration. Data are presented as mean ± SEM from three independent mitochondrial isolations, each analyzed with five technical replicates. Statistical analysis was performed using ordinary one-way ANOVA followed by Tukey’s multiple-comparisons test. * *p* < 0.05; ** *p* < 0.01; *** *p* < 0.001; **** *p* < 0.0001. Non-significant comparisons are not shown; Supplementary Figure S2: Mitoception alters the expression of fibrosis-associated, mitochondrial, and antioxidant genes at 6 h in patient-derived pulmonary fibroblasts. (A,B) Relative mRNA expression (2^−ΔΔCt^) of fibrosis-associated, mitochondrial, and antioxidant genes in normal fibroblasts (NF) (A) and diseased fibroblasts (DF) (B) following treatment with donor mitochondria (5 µg or 15 µg) for 6 h. Gene expression was normalized to β-actin. Data are presented as mean ± SEM from three independent biological replicates. Statistical analysis was performed using two-way ANOVA followed by Tukey’s multiple-comparisons test. * *p* < 0.05; ** *p* < 0.01; *** *p* < 0.001; **** *p* < 0.0001. Non-significant comparisons are not shown; Supplementary Figure S3: Effects of mitoception on the expression of mitochondrial dynamics-associated genes in patient-derived pulmonary fibroblasts. (A,B) Relative mRNA expression (2^−ΔΔCt^) of mitochondrial fusion (*OPA1* and *MFN1*) and fission (*DRP1* and *MFF*) genes in normal fibroblasts (NF) and diseased fibroblasts (DF) following treatment with donor mitochondria (5 µg or 15 µg) for 3 h and 24 h. Gene expression was normalized to β-actin. Data are presented as mean ± SEM from three independent biological replicates. Statistical analysis was performed using two-way ANOVA followed by Tukey’s multiple-comparisons test. * *p* < 0.05; ** *p* < 0.01; *** *p* < 0.001; **** *p* < 0.0001. Non-significant comparisons are not shown; Supplementary Figure S4: Mitochondrial transfer does not alter glycolytic activity. (A,B) Representative extracellular acidification rate (ECAR) traces of normal fibroblasts (NF) (A) and diseased fibroblasts (DF) (B) following treatment with donor mitochondria (5 µg or 15 µg) for 3 h. (C,D) Representative ECAR traces of NF (C) and DF (D) following treatment with donor mitochondria (5 µg or 15 µg) for 24 h. Data are presented as mean ± SEM from three independent biological replicates, each performed with five technical replicates. Statistical analysis was performed using ordinary one-way ANOVA followed by Tukey’s multiple-comparisons test. * *p* < 0.05; ** *p* < 0.01; *** *p* < 0.001; **** *p* < 0.0001. Non-significant comparisons are not shown; Supplementary Figure S5: Raw Western blot Data for Figure 1E; Supplementary Table S1: Clinical information of the human lung tissue samples used in this study.
