# Supplementary material for: Mitoception: A Novel Strategy to Alleviate Pulmonary Fibrosis
Source: Biology (Basel). 2026 Jul 9;15(14):1112. doi: 10.3390/biology15141112 (PMC13405580; doi:10.3390/biology15141112)
Supplement: Supplementary file 1 [file biology-15-01112-s001.zip › Sup Fig S5-Western Raw blots.pdf]

Raw Western blot Data for Figure 1E

FN1

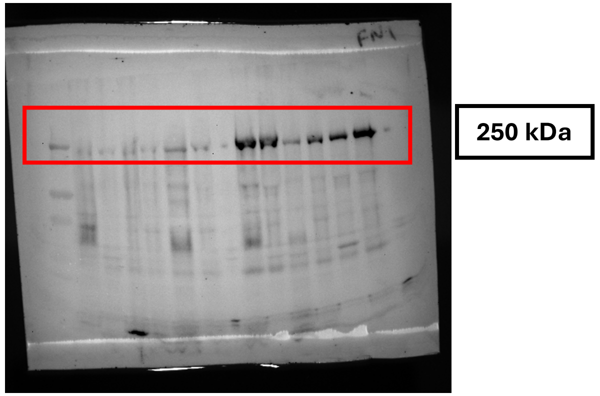

250 kDa

αSMA

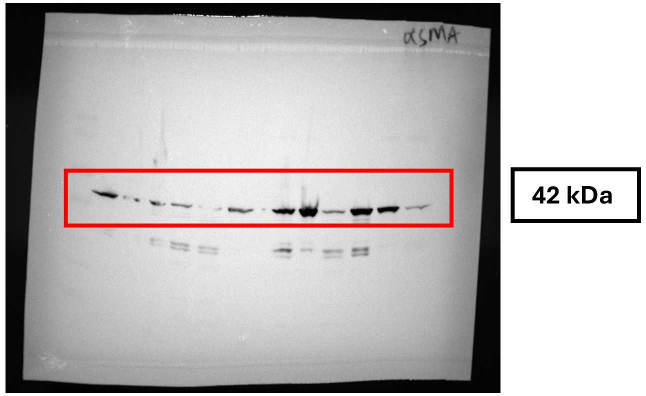

42 kDa

TGFβ

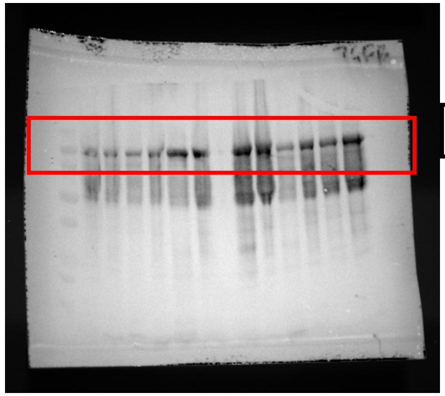

50-100 kDa

FN1-β actin

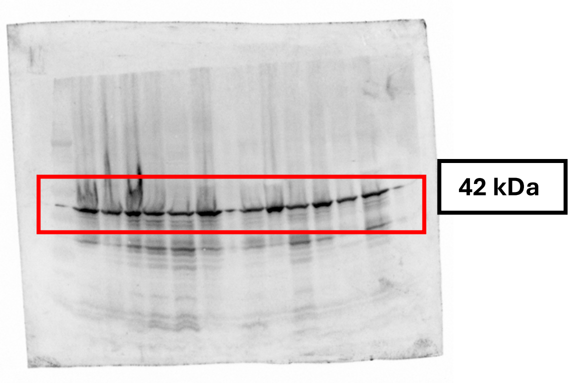

42 kDa

αSMA- β actin

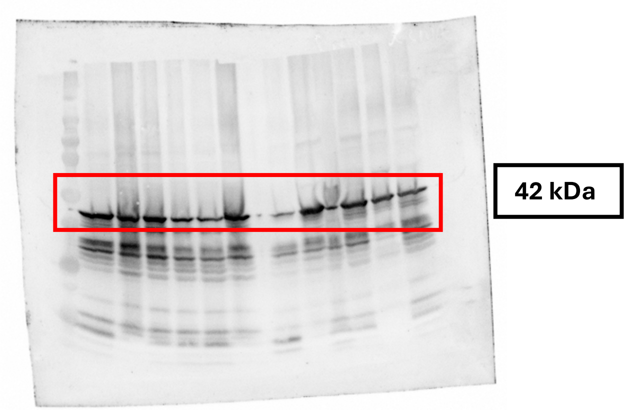

42 kDa

TGFβ- β actin

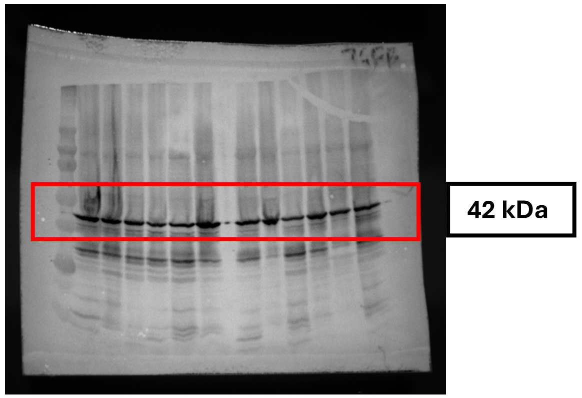

42 kDa
