# Supplementary material for: Mitoception: A Novel Strategy to Alleviate Pulmonary Fibrosis
Source: Biology (Basel). 2026 Jul 9;15(14):1112. doi: 10.3390/biology15141112 (PMC13405580; doi:10.3390/biology15141112)
Supplement: Supplementary file 1 [file biology-15-01112-s001.zip › Supplementary Table S1_Patient clinical info.pdf]

Supplementary Table S1. Clinical information of the human lung tissue samples used in this study

Sample	Location (if specified)	Diagnosis	Age at time of transplant	Sex
1	Lung	Fibrosis	64	Female
2	Lung	Fibrosis	62	Female
3	Lung	Fibrosis	56	Male
4	Lung	Fibrosis	69	Male
5	Lung	Fibrosis	63	Male
6	Lung	Fibrosis	64	Male
7	Lung	Normal	not listed	not listed
8	Right Lung	Normal	48	Male
9	Lung	Normal	56	Female
10	Lung	Normal	48	Female
11	Left Lung	Normal	49	Male
12	Right Lung	Normal	44	Male
