# Supplementary figures and images for: Mitoception: A Novel Strategy to Alleviate Pulmonary Fibrosis
Source: Biology (Basel). 2026 Jul 9;15(14):1112. doi: 10.3390/biology15141112 (PMC13405580; doi:10.3390/biology15141112)

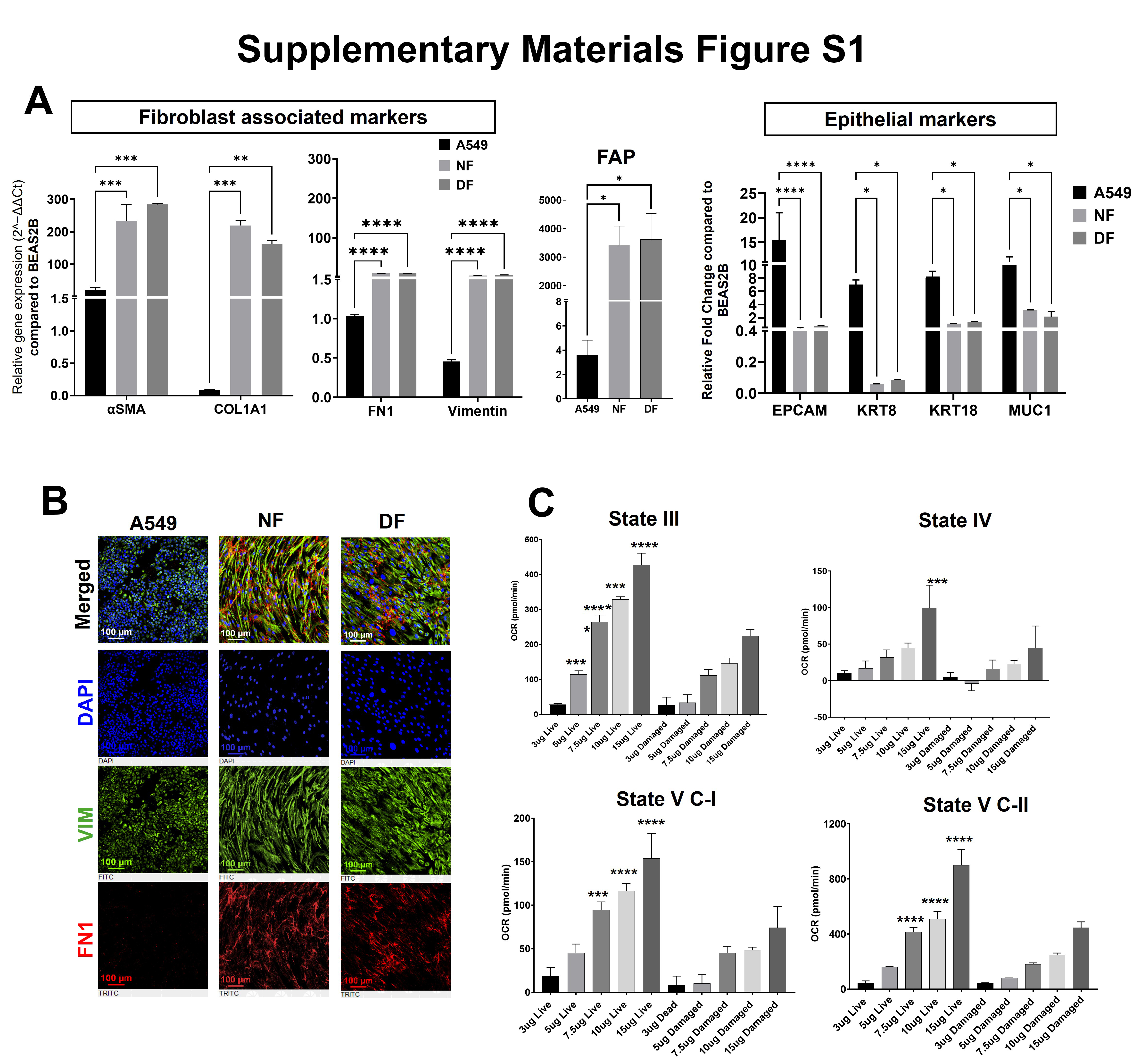

Supplement: Supplementary file 1 [file biology-15-01112-s001.zip › Sup Fig S1.jpg]

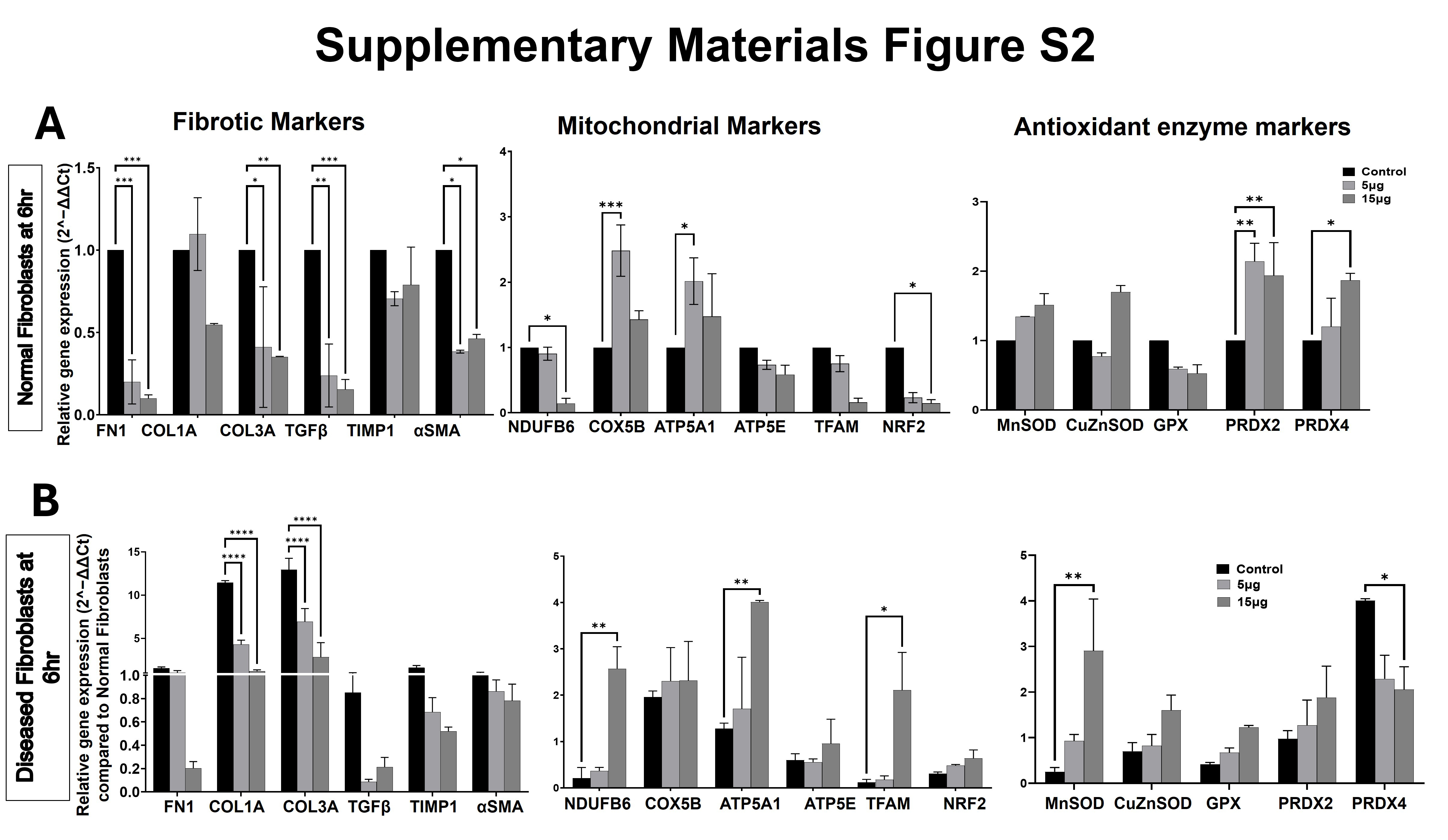

Supplement: Supplementary file 1 [file biology-15-01112-s001.zip › Sup Fig S2.jpg]

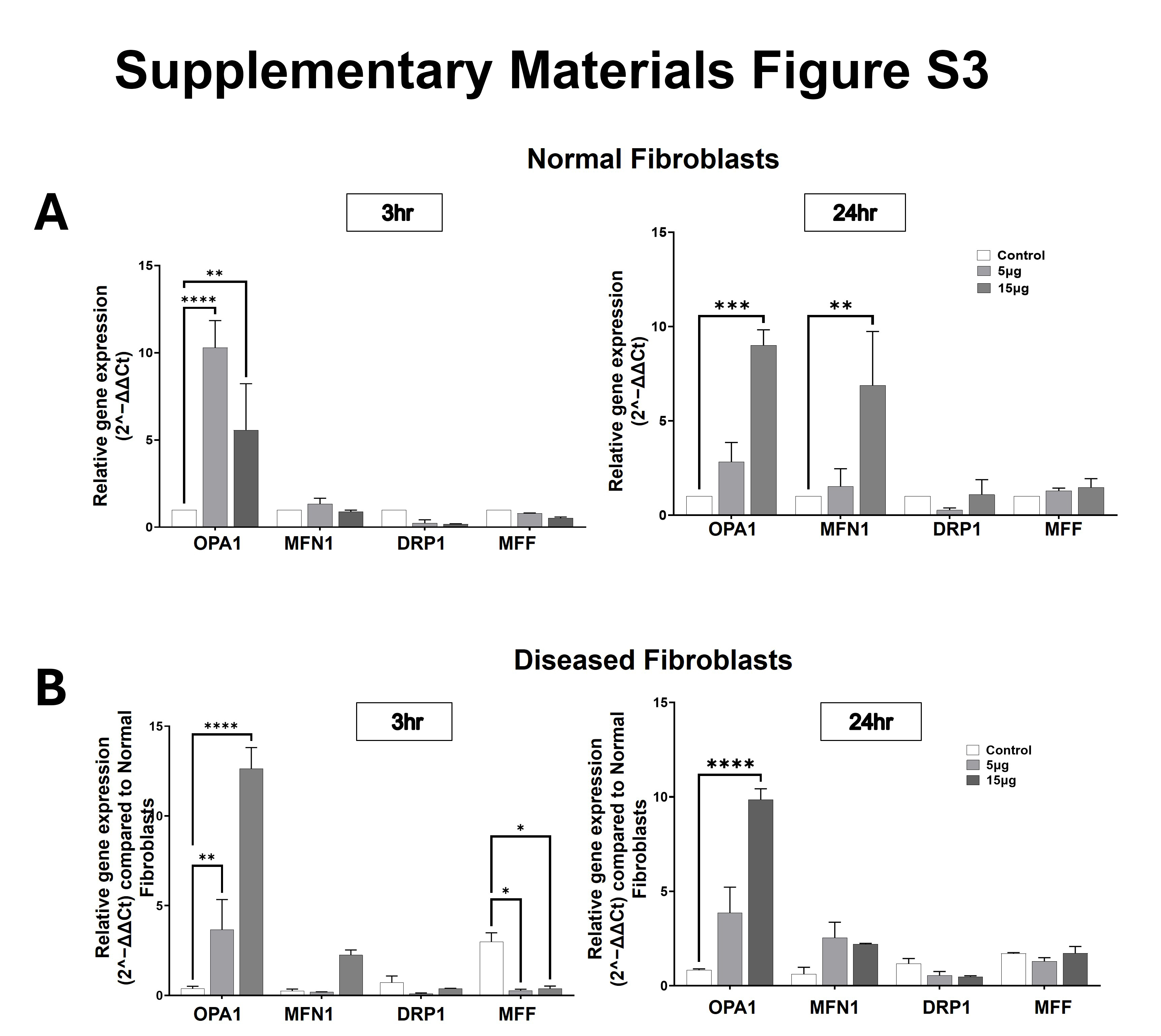

Supplement: Supplementary file 1 [file biology-15-01112-s001.zip › Sup Fig S3.jpg]

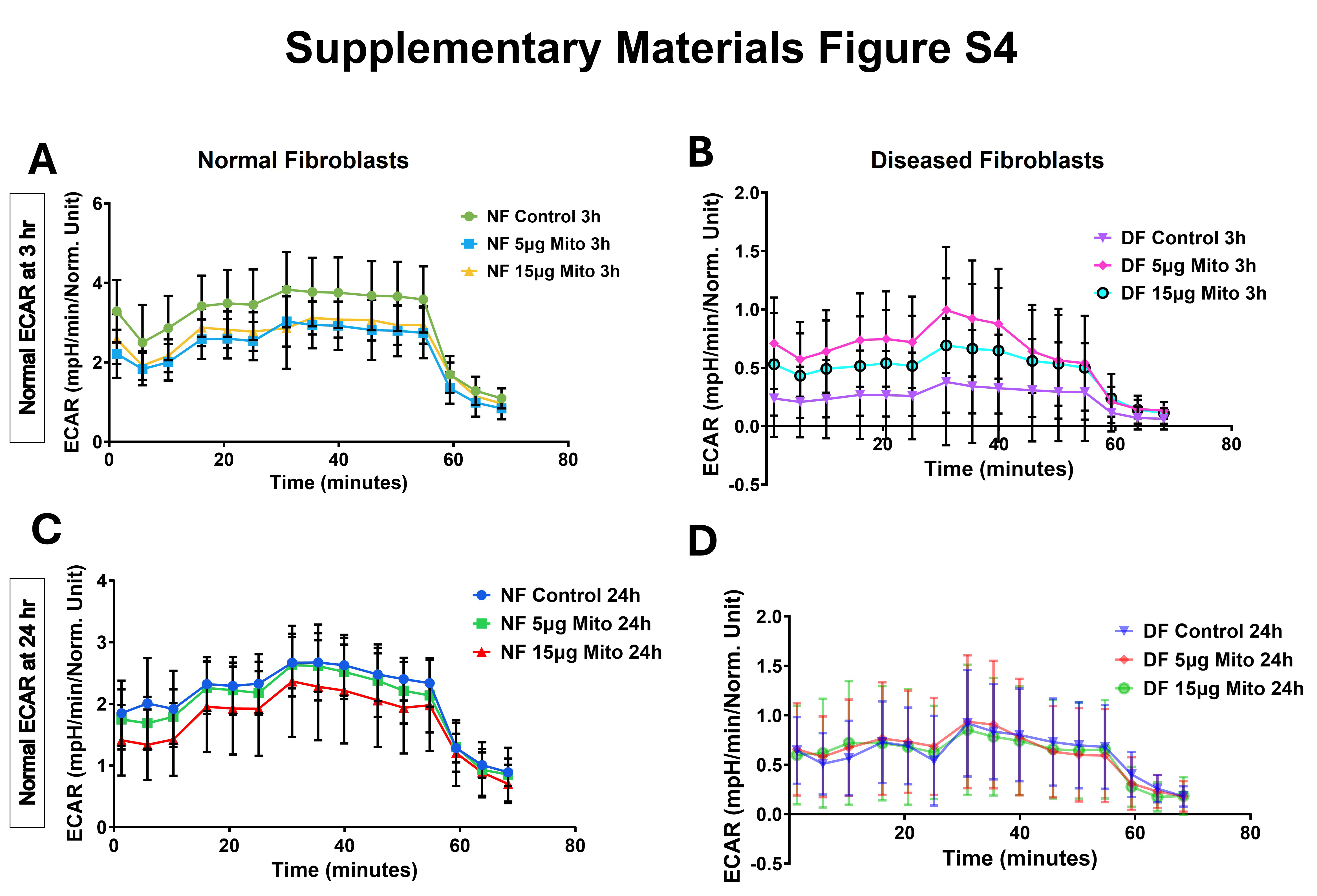

Supplement: Supplementary file 1 [file biology-15-01112-s001.zip › Sup fig S4.jpg]
